# Medical Education and Charity

**Published:** 1898-04-23

**Authors:** 


### The Hospital

A Journal of

The Medical Sciences and Hospital Administration.

Vol. XXIV.—No. 604.] Edited by Sir Henry Burdett, K.C.B., and Solomon C. Smith, M.D., M.R.C.P. [April 23, 1898.

### MEDICAL EDUCATION AND CHARITY.

Everybody must sympathise with the appeal which the Hon. Sydney Holland is making on behalf of the London Hospital, and we can only hope that that appeal will bring in all the money which is so urgently required for the largest of our voluntary hospitals. But in his letter to the *Times*, Mr. Holland states that £2,000 a year of the general funds of the hospital have been voted by the Committee towards the cost of maintaining the Medical School. This is a new departure, and one which we feel is neither in the interests of medical education nor just to the hospital, which is so badly in want of additional funds to enable it to grapple successfully with the demands made upon its resources by the sick poor resident in one of the poorest of metropolitan districts. Before considering the educational aspects of the question thus raised, we may point out that medical students belong to a class of the population who are perfectly well able to defray all the cost entailed by their proper instruction and training for the profession of medicine. The fees charged at the London Hospital for the course of medical study, when compared with the fees which have to be paid by parents who put their sons into other professions, are very small. There are, Mr. Holland states, 350 medical students attached to the London Hospital at the present time, so that an extra £10 a year each would not only provide the £2,000 extra revenue which is required by the medical school, but would give the school authorities an ample balance to enable them to increase the facilities for medical study which they offer as an inducement to parents to send their sons to that institution. We are perfectly certain, if it is made clear to parents who have sons desirous of entering the medical profession, that if they are not careful a portion of the cost of the education of their children may be defrayed out of the voluntary offerings of the public for the relief of the poor who are sick and suffering, that in making their choice of a medical school they will take care not to send their children to any institution which would lay them open to the charge that they were in any sense the recipients of charity. We must, therefore, place on record our protest against the policy of devoting any portion of the charitable revenues of a voluntary hospital to maintain its medical school; and at the same time express a hope that the whole question will be reconsidered where such grants may have been made, with a view to their immediate discontinuance.

That there is in fact no justification for such a grant of charitable funds for the purposes of a medical school will be made clear by the following facts, illustrating the course of medical study in London at the present time. It is a fact that the expense of running a medical school does not lie in the teaching of medicine and surgery, but in the elaborate appliances required for the proper teaching of the scientific preliminaries which occupy the first two years of the student's career. During these two years, or more, the student need never enter the hospital; and he would, probably, be far better taught in some great central school, fitted with the most perfect appliances and provided with the most skilled teachers obtainable, than he is under the present system, according to which each hospital tries to provide a complete set of laboratories and dissecting rooms, of professors and of demonstrators, however small may be the number of students who may attend the classes. This is where the money goes, not in the teaching of medicine and surgery. By all means let every hospital provide, not merely hospital beds, but the most complete laboratories and the most perfect appliances for the investigation of disease. Let there be no stint in these matters, and let the students, in using these appliances for the benefit of the patients, get out of them every bit of learning and experience that they can. In return for what the students do for the hospital let the hospital take care that the treatment of the patients and the investigation of their ailments are carried out under such conditions that students shall be enabled to get the full reward of their labour in the form of knowledge and experience.

But as regards scientific training, as regards that college teaching by which alone the student can be fitted for his work in the wards, that, surely, is for his own personal benefit, and for that he should himself pay. As a fact there is no reason why he should not pay, nor, indeed, has he asked to be excused. The largest medical school in London is the one that charges the highest fees, and if any metropolitan hospital medical school cannot give an equally good education for a smaller sum, which is very possible, its authorities should either raise their fees or adopt some form of combination with other schools to get rid of the suicidal wastefulness which is involved in each hospital keeping up a school for the teaching of subjects which have nothing to do with the hospitals at all.

We have very little doubt that it is under the glamour of the idea that a great school is absolutely essential to a great hospital, that students must be attracted, that a talented staff must be drawn together by the offer of teaching facilities, and that the money given to the school is but a payment for advantages obtained by the patients in virtue of the connection of the hospital with the school—advantages which could not otherwise be obtained—that the managers of certain hospitals have occasionally been led to subsidise their schools.

We think, however, that this is an entirely false view of the situation. As a matter of fact, the student is by no means essential to the hospital, however advantageous his presence may be. This is proved by the knowledge that there are many large hospitals of undoubted efficiency where there are no students at all. But the hospital is absolutely essential to the student. By no stretch of imagination can we conceive of a student obtaining an efficient medical education without a hospital. How then, it may fairly be asked, has such a hospital as the London ever been led to grant a couple of thousand a-year to its medical school, and thus make a present of, say, £20 worth of education free gratis and for nothing to every student who enters there?

The answer is that the whole thing has arisen from the undue multiplication of schools in the metropolis, from their unreasonable competition with each other, and from the lamentable absence of business capacity and power of combination, which has allowed each school to endeavour to be complete in itself, and to maintain that it is equal to its neighbours in every branch of knowledge.

The remedy, then, is to be sought in a federation of several metropolitan medical schools for the purpose of providing the student with the best instruction in the subjects which occupy the first two years of his medical career. This could readily be done on the basis already indicated, and the sooner it is taken in hand the better it will be for the interests of all concerned. As we have shown, this improved system would in no way interfere with the present high standard of medical and surgical work to be found in our great hospitals. There the clinical instruction must always be given, and there practical medicine and surgery must be taught, so that our great school hospitals must continue to attract the most highly qualified medical staff, whose teaching the medical student will gladly avail himself of, especially when he is guaranteed—as he would then be guaranteed—that in seeking it he is incurring no risk of being charged with having deprived the poor of money given for their relief and medical treatment.

